# Exploring the World of Membrane Proteins: Techniques and Methods for Understanding Structure, Function, and Dynamics

**DOI:** 10.3390/molecules28207176

**Published:** 2023-10-19

**Authors:** Imad Boulos, Joy Jabbour, Serena Khoury, Nehme Mikhael, Victoria Tishkova, Nadine Candoni, Hilda E. Ghadieh, Stéphane Veesler, Youssef Bassim, Sami Azar, Frédéric Harb

**Affiliations:** 1 Faculty of Medicine and Medical Sciences, University of Balamand, Tripoli P.O. Box 100, Lebanon; imad.boulos@std.balamand.edu.lb (I.B.); joy.jabbour@std.balamand.edu.lb (J.J.); serena.khoury@std.balamand.edu.lb (S.K.); nehme.mikhael@std.balamand.edu.lb (N.M.); hilda.ghadieh@balamand.edu.lb (H.E.G.); youssef.bassim@balamand.edu.lb (Y.B.); sami.azar@balamand.edu.lb (S.A.); 2 CNRS, CINaM (Centre Interdisciplinaire de Nanosciences de Marseille), Campus de Luminy, Case 913, Aix-Marseille University, CEDEX 09, F-13288 Marseille, France; victoria.leoni@univ-amu.fr (V.T.); nadine.candoni@univ-amu.fr (N.C.); stephane.veesler@cnrs.fr (S.V.)

**Keywords:** membrane proteins, electrophoresis, X-ray crystallography, nuclear magnetic resonance, computational techniques, artificial intelligence, atomic force microscopy, cryo-electron microscopy

## Abstract

In eukaryotic cells, membrane proteins play a crucial role. They fall into three categories: intrinsic proteins, extrinsic proteins, and proteins that are essential to the human genome (30% of which is devoted to encoding them). Hydrophobic interactions inside the membrane serve to stabilize integral proteins, which span the lipid bilayer. This review investigates a number of computational and experimental methods used to study membrane proteins. It encompasses a variety of technologies, including electrophoresis, X-ray crystallography, cryogenic electron microscopy (cryo-EM), nuclear magnetic resonance spectroscopy (NMR), biophysical methods, computational methods, and artificial intelligence. The link between structure and function of membrane proteins has been better understood thanks to these approaches, which also hold great promise for future study in the field. The significance of fusing artificial intelligence with experimental data to improve our comprehension of membrane protein biology is also covered in this paper. This effort aims to shed light on the complexity of membrane protein biology by investigating a variety of experimental and computational methods. Overall, the goal of this review is to emphasize how crucial it is to understand the functions of membrane proteins in eukaryotic cells. It gives a general review of the numerous methods used to look into these crucial elements and highlights the demand for multidisciplinary approaches to advance our understanding.

## 1. Introduction

All eukaryotic cells are surrounded by a cell membrane that separates and protects their internal environment from the extracellular environment. This membrane is constituted of phospholipids, cholesterol, glycolipids, and proteins. Membrane proteins are encoded by 30% of the human genome [1,2]. The proteins exist in a variety of shapes and sizes [3] and can be classified according to their positions in the cell membrane into 3 categories: integral proteins, extrinsic proteins and intrinsic proteins. The integral proteins span the lipid bilayer and are stabilized within the membrane by hydrophobic interaction and within the aqueous compartments by hydrophilic interactions. The extrinsic proteins bind to the outer hydrophilic leaflet of the bilayer and interact with other proteins or lipids via electrostatic interactions, whereas the intrinsic proteins are imbedded within the lipid bilayer, interacting only with its hydrophobic portion [4]. The membrane proteins play vital physiological roles necessary for the survival of the cells, according to which they can be further classified into channels, transporters, pumps, enzymes, cytochromes P450 (CYPS), G-proteins-coupled receptors (GPCRS) and many more. The channels and the pores control the permeability as well as the exchange between the cell and its surroundings [5]. Ion channels control the passage of several ions in and out of the cells, regulating several physiological functions such as electrical signaling in the heart and nervous system, fluid secretion in the lungs, kidneys, gastro-intestinal (GI) tract, hormones secretion, immune response, bone remodeling and tumor cell proliferation. A wide number of diseases affecting the cardiovascular, nervous, metabolic, respiratory as well as other systems, are related to ion channel dysfunction. Therefore, ion channels are being targeted by many pharmaceutical drugs, aiming to cure ion-channels related disorders [6]. Membrane-embedded transporters are another type of membrane proteins that essentially contribute to the uptake of nutrients by the cells and to the removal of any unwanted substances, while conserving the physiological concentrations of the molecules in the cells. Certain diseases such as obesity and cancer can be traced back to defects in membrane transporters. Therefore, the development of therapeutic drugs that target them relies partially on understanding their mechanism of function. Another important function carried by the transporters is the delivery of drugs into the cells or across membranes or barriers, such as the blood-brain barrier, which emphasizes on the importance of their role in drug therapies [7]. The Na^+^/K^+^ pump is also an example of membrane proteins that transport sodium and potassium against their concentration gradient. It is involved in several physiological mechanisms related to the cardiac and nervous systems among others, and in the activation of signaling pathways that regulate cell growth. This pump is the target of several researches that aim to understand the physiological mechanism behind its function, leading to the development of specific therapeutic drugs [8]. Membrane bound enzymes constitute a large portion of the intracellular enzymes. They are involved in translocation, information transfer, and in acting on neighboring proteins and molecules. Their diverse functions make them the target of half of the medical drugs [9]. GPCRs are receptors located on the plasma membrane. When a G-protein binds to its receptor, it initiates downstream signaling cascades, that lead to the activation of biochemical pathways. They are present on various cell types, in multiple tissues and organs, where they regulate diverse cell and tissue specific physiological functions, such as cell signaling, cell division and proliferation, signal mediation from receptor tyrosine kinases and more. Defects in GPCRS are associated with impaired motor coordination secondary to cerebellar development, defective platelet activation, cardiac malformation, craniofacial defects, and hyperparathyroidism [10], which makes their study crucial for the understanding of disease mechanisms as well as for the development of the necessary therapeutic agents. Another example is the CYPs which are monooxygenases, involved in several reactions, that end up either activating prodrugs or enhancing their clearance and excretion by the kidneys. Comprehending their function is especially crucial in the study of cancer drugs, since the catalytic activity of CYPs enhances drug clearance, and reduces their efficacy as a result [11]. The wide range of membrane proteins and their participation in numerous essential physiological processes [1,12], coupled with the association of their malfunction with various diseases [2], as well as their involvement in viral and bacterial infections, virulence, and antimicrobial resistance [13], highlights the critical significance of studying their structures and functions. This research is particularly crucial for the development of drugs that specifically target membrane proteins [12], aiming to enhance therapeutic advancements. 

The function of membrane proteins depends on several factors including their conformation, their specific location in the lipid bilayer, and their specific interaction with other proteins and lipids [1]. All these factors should be considered while studying protein function. The protein function is strongly linked to its structure [7]. The native structure of a protein is determined by the sequence of amino acids in its polypeptide chain, and is stabilized by an interaction between several forces that include covalent bonds, hydrogen bonds, and other attractive/repulsive forces (e.g., electrostatic forces, Van der Waals forces, etc.) [14]. Proteins acquire new functions due to evolutionary changes in their amino acid sequences. Certain mutations in specific amino acid sequences of proteins, that translate into a change in structure, consequently translate into a change in function, which can be a loss of function, a change of function or a gain of function in some cases [7]. While identifying the protein structure is essential, it may not provide a complete understanding of its molecular function. To gain deeper insights into the mechanisms of protein function, it is crucial to ensure the preservation of correct folding in membrane proteins during extraction and studying them within an environment that closely mimics their physiological conditions. By taking these factors into account, a more comprehensive understanding of the mechanisms underlying their function can be achieved [15]. Experimentally determining the structure of membrane proteins has been proven to be more difficult compared to that of other proteins [16]. Membrane proteins are insoluble in water, so they need to be solubilized using detergents that denature them, whereby changing their natural 3D conformation [17]. Transmembrane proteins are physiologically present in a lipid environment and interact with its components, which is required for their function [18]. Solubilizing the membrane proteins during extraction changes their native environmental conditions and results in an irreversible disruption of their structure [19]. Similarly, a large amount of membrane proteins is required to be able to characterize them, along with other practical problems that accompany the purification techniques [2]. 

**Figure 1:**
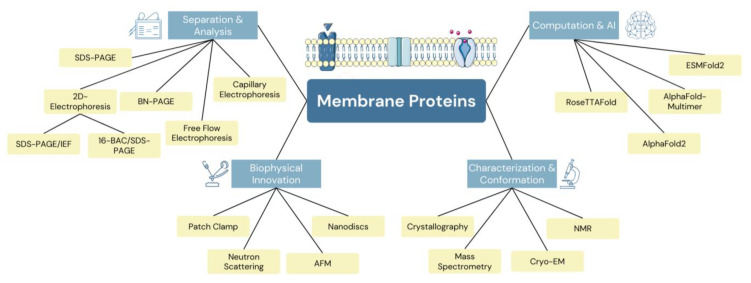
Graphical summary of the common techniques used for the detection and characterization of membrane proteins.

Thus, the objective of this review is to comprehensively explore the various techniques and methods (summarized in Figure 1) employed in the study of membrane proteins with the aim of enhancing our understanding of their structure, function, and dynamics. By examining a range of experimental and computational approaches, we seek to provide insights into the intricacies of membrane protein biology. The review will encompass an array of techniques, including but not limited to electrophoresis, X-ray crystallography, cryogenic electron microscopy, nuclear magnetic resonance spectroscopy, biophysical techniques, and computational methods. Through an evaluation of these diverse methodologies, we aim to highlight their contributions, limitations, and potential synergies in advancing our knowledge of membrane proteins. This comprehensive assessment will serve as a valuable resource for researchers, guiding them towards the most effective strategies to unravel the complexities of these crucial biomolecules.

## 2. Techniques and Methods Used for Protein Analysis

### 2.1. Separative Techniques

#### 2.1.1. Electrophoresis

Electrophoresis is the separation of biological compounds, under an electric field, such as DNA, RNA, and proteins based on their charge and size [20]. This technique is vastly utilized in laboratories for multi-disciplinary fields such as forensic sciences [21], conservation biology [22,23], molecular biology [24], and medicine [25,26]. There are numerous types of electrophoresis methods with different components, however, when it comes to the separation of membrane proteins, some electrophoreses methods have been deemed the convention while others fall short because of their limitations sine they can present the difficulty in studying hydrophobic proteins, or being unfit for the analysis of oligomerized protein, or even time consuming and need specialized equipment, or even because of being inefficient.

#### 2.1.2. Sodium Dodecyl-Sulfate Polyacrylamide Gel Electrophoresis or SDS-PAGE

Sodium dodecyl-sulfate polyacrylamide gel electrophoresis is employed to separate membrane proteins based on their molecular weight [27]. With the use of SDS lysis buffer and polyacrylamide gel, the factors of charge and shape no longer contribute or influence this test’s findings, as SDS is a solubilizing detergent that denatures the proteins, coating them with a negative charge along their length [28]. Hence, all proteins will share the same charge and through the denaturing step will also have the same shape. This makes molecular weight the sole differentiating factor. With stains such as colloidal Coomassie blue [29], the migration can be observed and can be compared with a reference molecular ladder to determine molecular weight. This was implicated in the purification peripheral membrane protein FAM92A1 [30]. However, this method is rarely used on its own. It is coupled with other techniques, such as the Enzyme-mediated activation of radical sources (EMARS) reaction which found host cell membrane proteins near the spike protein attachment site of SARS-CoV-2 [31] and mass spectrometry for the study of membrane protein present in the extracellular vesicles of carbapenem-resistant Klebsiella pneumoniae [32], and in complementarity with Two-dimensional gel electrophoresis coupled to mass spectrometry (2DE-MS) [33].

### 2.2. Dimensional Electrophoresis (SDS-PAGE/Isoelectric Focusing)

In proteomics, molecular biology, and dimensional electrophoresis, this technique, commonly dubbed 2D electrophoresis, is a flexible and frequently used method. It combines the isoelectric focusing (IEF) and SDS-PAGE separation techniques to enhance resolution and separation of complicated protein mixtures. Moreover, this method is useful for detecting protein post-translational modifications (PTMs) and differentiating protein isoforms. Thus, this method employs two approaches: the first is the isoelectric focusing that incite separation based on isoelectric points of the proteins (pI), and the second is the conventional aforementioned SDS-PAGE to account for their molecular weight [34]. In isoelectric focusing (IEF), a pH gradient is established using immobilized pH gradient (IPG) strips, where proteins are placed in a gel matrix and subjected to an electric field. The proteins migrate through the gel until they reach the pH region that matches their respective pI values, at which point they become electrically neutral and stop migrating. In the second approach, these proteins are denatured using sodium dodecyl-sulfate (SDS) and subsequently separated on SDS-PAGE based on their molecular size. After arranging the strips on the gel and applying an electric charge, protein migration begins from the IPGs to the gel, leading to the separation process. The use of stains such as Coomassie Blue and silver nitrate helps visualize protein migration path [35]. This technique was proven useful in the study of erythropoietin analogs Epotin, Hemax and Jimaixin identifying differences in their structure from main drug Eprex [36]. This has also been used to study membrane proteins of several bacteria: *E. coli* [37], *Salmonella typhimurium* [38], *Edwardsiella tarda* [39], *Shigella flexneri* [40], *Leptospira interrogans* [41], *Riemerella anatipestifer* [42], and *Campylobacter jejuni* [43], with more recently adding *Klebsiella pneumoniae* to the list with the addition of zwitterionic detergent Zwittergent Z 3–14^®^ to further aid solubilization [44].

#### 2.2.1. Dimensional Electrophoresis (16-BAC/SDS-PAGE)

This technique has been adapted to tackle the shortcomings of the IEF/SDS-PAGE. It enhances the solubility and recovery of the highly hydrophobic membrane proteins in comparison with the conventional 2D IEF/SDS-PAGE, as well as the low-pH environment which has been aided in the preservation of the unstable methylation of basic proteins [45]. This has been used to successfully separate membrane proteins of Plasmodium falciparum-infected erythrocytes, with the help of cationic detergents such as benzyldimethyl-n-hexadecylammonium chloride (16-BAC) [46]. It has also served in the electrophoresis of mitochondrial membrane proteins of low molecular mass [45]. With the use of this procedure, protein-rich samples may be analyzed at high resolution. 

#### 2.2.2. Blue Native PAGE

Blue Native PAGE, or BN-PAGE, is a strategy used to study proteins in their native biological state. This technique particularly has a great value in studying membrane proteins, as it allows the analysis of membranes in subcellular segments, without subjecting them to denaturing agents and altering their biological composition. These segments are isolated and suspended in carbohydrate or glycerol-rich buffer, then frozen and solubilized with a non-harmful detergent to undergo polyacrylamide gel electrophoresis [47]. Thylakoid membrane complexes of Arabidopsis chloroplasts were studied and separated using BN-PAGE, allowing the observation of their dynamic states [48]. This routine opens doors for vast utilization in the field of proteomics, especially in the domain of membrane complexes. For instance, it was used in the study of stability of mitochondrial respiratory complexes at different temperatures [49]. Similarly, the separation of heart mitochondrial respiratory complexes in mouse models was performed with BN-PAGE, to then be coupled with mass spectrometry [50].

#### 2.2.3. Capillary Electrophoresis 

This electrophoresis technique separates substances based on the correlation between the speed of an ion in motion and its charge-to-size ratio [51]. It offers excellent separation efficiency and high resolution for the analysis of a wide range of compounds, including small ions, polar molecules, proteins, peptides, and nucleic acids. Capillary electrophoresis (CE) is based on the principle of electrophoretic migration of analytes through a narrow capillary filled with an electrolyte solution, driven by an electric field. The separation is achieved by exploiting differences in the analytes’ electrophoretic mobility, which is influenced by their charge, size, and shape. This technique provides numerous advantages, such as short analysis time, low sample and reagent consumption, and the ability to analyze complex samples. Moreover, CE can be coupled with various detection methods, including UV-Visible spectroscopy, fluorescence, mass spectrometry, and electrochemical detection, enhancing its versatility and applicability. This method has been used to study the membrane proteins of bacterial strains of Pseudomonas upon antibiotic treatment [52], as well as to quantify membrane proteins of Chinese hamster ovary cells by GFP tagging [53]. Extracellular vesicle membrane protein CD63, a member of the tetraspanin family, was targeted using capillary electrophoresis immunoassay coupled with laser-induced fluorescence [54], further proving its versatility. 

#### 2.2.4. Free Flow Electrophoresis

Free flow electrophoresis (FFE) is an innovative and versatile separation technique that has emerged as a powerful tool in various scientific disciplines. It offers efficient and rapid separation of complex mixtures of biomolecules, such as proteins, nucleic acids, and cells, based on their charge, size, and surface properties. FFE utilizes thin liquid films that does not denature the membrane proteins [55,56]. It entails the centrifugation of cell lysates to obtain an intricate mixture of membranes operates, then subjecting the sample to an electric field while it flows through a thin flow channel, enabling the separation of analytes in a continuous manner [55]. This technique provides unique advantages, including high separation efficiency, minimal sample loss, and the ability to process large sample volumes. FFE has found applications in diverse fields, including proteomics, genomics, drug discovery, and biotechnology. This technique has been successfully used for the isolation of plant organelles such as the mitochondria and plasma membranes [56,57]. It was also used to study the interactions of membrane protein (AQP0) and calmodulin [58].

To enhance clarity and comprehension, we have condensed the information from this section into Table 1 below.

### 2.3. Techniques for Characterization and Structural Analysis

Characterization and structural analysis of a membrane protein in their native form is crucial for identifying the properties, understanding their biological mechanism as well as essential in drug discovery. Although many challenges are faced when identifying these integral membrane proteins, numerous techniques have been used in order to analyze them: Crystallography, Cryogenic Electron Microscopy and Nuclear Magnetic Resonance (NMR) spectroscopy. Each of these has its own advantages and disadvantages.

#### 2.3.1. X-ray Crystallography

Crystallography is one of the most famous techniques used to identify 3D structure of proteins [78]. It is based on the formation of molecular crystals and then with using one of several methods to determine the structure of the protein. Each of these methods have its own advantages and limitations, for instance, we can use X-ray crystallography, electron crystallography or neutron crystallography [79,80]. X-ray crystallography is the primary procedure utilized to analyze and attain a comprehensive understanding of protein structures at near atomic or even atomic resolution [81]. It relies on the generation of electron density maps that illustrate the shape and structure of the crystallized macromolecules. The crystals should have adequate dimensions for the X-ray diffraction measurements in order to obtain clear pictures [82]. 

This technique has been used for decades and has undergone a lot of advancements, but still suffers from many limitations especially in the crystallization phase [83]. For instance, the crystallization of membrane proteins takes a lot of time and effort [84], in addition to the inability to form large, well-ordered crystals [83]. Many crystallization techniques were developed to overcome this constraint, such the use of lipidic cubic phase (LCP) which provides a more native-like environment for crystallizing membrane proteins [85,86]. This has led to the identification of many structures like the channel rhodopsin light-gated cation channel [87]. 

Furthermore, the X-ray radiation that the crystals are exposed to is an important challenge in X-ray crystallography, compromising the quality of the collected data. This have been overcome by formation of large crystals, but this isn’t always feasible without compromising the quality of the crystals [88]. A new more improved method is now being used called X-ray free electron lasers (XFEL) which deliver extremely short and intense X-ray pulses, these pulses are so brief that they can minimize radiation damage [89]. XFELs are also effective in the advancement of Serial Femtosecond Crystallography (SFX), which is a revolutionary technique for studying the structure of membrane proteins. In SFX, crystals are continuously delivered into the XFEL beam, and each crystal is destroyed after a single X-ray pulse. This approach significantly reduces the effects of radiation damage and enhances data quality [90]. For instance, using Time-Resolved Serial Femtosecond Crystallography (TR-SFX) researchers were able to unveil the dynamic structures of diverse microbial rhodopsins. and that these rhodopsins serving as either pumps or channels, share common characteristics in their conformational changes triggered by light excitation [91].

Another limitation is the failure to experimentally visualize hydrogen atoms. As a matter of fact, the visualization of hydrogen atoms in X-ray crystallography of proteins is essential for accurate atomic modeling because it enables the validation and improvement of the atomic model, offers insights into the protein’s hydration shell, and promotes the rational structure of ligands interacting with the protein’s active site. This can be overcome by using neutron protein crystallography to visualize and identify the positions of hydrogen and deuterium [92,93]. To demonstrate this, researchers used neutron crystallography to determine the hydrogen atom positions within the lecb/Ca/fucose complex situated in the membrane of *P. aeruginosa* [94]. Furthermore, this technique has been applied to uncover the hydrogen atom arrangement in metalloproteins, helping in the comprehension of diverse protonation states and intricate hydrogen-bonding network [93]. The challenges of low solubility, post transcriptional epigenic modification, and insufficient detection of chemical heterogeneity also pose limitations for X-ray crystallography [78]. For instance, the existence of multiple conformational states within a sample may inhibit the formation of crystals, and even if heterogeneous molecules were arranged in a crystal lattice, X-ray crystallography can only identify and differentiate heterogeneity [78]. 

#### 2.3.2. Cryogenic Electron Microscopy

Cryo-electron microscopy (Cryo-EM) is a technique that involves vitrification of the purified protein sample with liquid nitrogen or liquid ethane. This leads to the creation of a thin, amorphous ice layer containing the purified protein, enabling direct visualization with a low-dose transmission electron microscope (TEM) operating at liquid nitrogen temperature [95,96]. Cryo-EM can be used to determine the structure of isolated biomolecular complexes, covering a wide range of molecular mass, spanning from small proteins to large viruses and cells [96]. It commonly provides insights into proteins at a coarse level of detail, around 4 Å. In recent years, the Cryo-EM witnessed a “resolution revolution” in EM through continuing progress enabling scientists to study some proteins with far greater resolution [97]. For example, researchers were able to acquire the structures of various protein complexes found in the membrane of *P. falciparum* at near-atomic resolution (3.2 Å) [98], PA28γ was imaged at 2.82 Å in resolution [99], Apoferritin reached 1.25 Å [97], and GABAa R was resolved to 1.7 Å [100].

In addition, many challenges, which used to pose problems with other techniques especially in the analysis of the structure of membrane proteins, have been overcome. Cryo-EM is now able to distinguish heterogeneous complexes computationally [101], either by focusing on the homogeneous parts of the complex [102], or by doing single-particle cryo-EM and collecting the data of an ensemble of a heterogeneous ensemble of subunits [103,104]. The recent “resolution revolution” of cryo-EM has made it easier to identify single pass transmembrane receptors (SPTMRs) like GPCR. This advancement has been very helpful in characterizing GPCR proteins. Furthermore, the breakthroughs aided by cryo-EM’s high-resolution, provide unmatched insights into the molecular interactions that govern SPTMR activity [105]. Despite cryo-EM’s ongoing challenges in visualizing small proteins with a molecular weight below 100 kDa [106], the structural analysis of GPCRs (~40–50 kDa) remains at the limit of cryo-EM’s. However, cryo-EM is able to provide high-resolution structural insights into GPCR complexes with heterotrimeric G-proteins [107]. With structural identification of the transmembrane peptides, containing the allosteric sites of GPCRs, it becomes possible to design specific peptides that could potentially disrupt and inhibit GPCR activity [108].

Cryo-EM has been particularly useful in determining bacteria’s multidrug efflux transporters. It has successfully revealed the structural details of bacterial RND transporters, offering prospects for designing structure-guided drugs targeted against the distinct configuration of these transporters, intended for combating both multidrug-resistant (MDR) and extensively drug-resistant (XDR) bacteria [109]. A representative case involves the determination of RE-cmeb’s structure, a constituent of a specific bacterial multidrug efflux pump in *Campylobacter jejuni*, using single-particle cryo-EM. The achieved resolution falls within the range of 3.08 Å to 3.39 Å [110]. 

Another cryogenic technique called Cryo-electron topography (Cryo-ET) can be used to visualize membrane proteins. This technique has the advantage on Cryo-EM is that it surpasses the need of the purification and extraction process. Cryo-ET is able to study membrane proteins and visualize them at a 3D level within the context of intact cellular membranes [111]. However, the resolution of Cryo-ET is still limited unlike Cryo-EM which as discussed before is now able to give the structure of membrane proteins at very high resolutions [112]. 

#### 2.3.3. Nuclear Magnetic Resonance Spectroscopy

Nuclear Magnetic Resonance (NMR) is a highly versatile and powerful technique used across numerous scientific fields including biology. It also has vast applications in medical and clinical settings for disease detection [113,114]. But NMR is mainly applied in order to acquire 3D structures of membrane proteins at an atomic resolution in their native lipid bilayer [115], or in reconstructed near-native composition of the lipid bilayer [116]. It is based on the detection of different energy levels after applying a magnetic field on an atom which has non-zero nuclear spins [117]. Not all nuclei are NMR active, the most important are ^1^H NMR [113] and ^13^C NMR [118,119], but there are also other types such as ^31^P NMR [118] and ^19^F NMR [120].

Unlike other methods, such as cryo-EM and X-ray crystallography [14,121] that only gives static structures of proteins and encounter limitations when analyzing intrinsic disordered proteins, NMR emerge as a powerful technique for the analysis and characterization of these disordered protein. It is highly sensitive to the conformational dynamics of these complexes [122], and the measurement of nuclear spin relaxation using NMR enables the study of conformational dynamics [123]. 

There are 2 main types of NMR:

- Solution NMR is a technique that is important to study proteins in solution. It’s used to study membrane protein folding, interactions, conformational changes, and internal mobility, in addition to ligand-substrate interactions [124]. One of its main limitations is size, as it is particularly useful for studying small to medium-sized proteins. In the past decades, it went form only detecting 10 kDa proteins in the 1980s to around 25–35 kDa in the mid-1990s [117]. Recent advancements in high-field magnets and cryogenic probes, together with new sample preparation protocols and transverse relaxation-optimized methods, have pushed solution NMR protein size limitations to reach almost 100 kDa in some rare instances [117]. For instance, researchers were able to detect conformational changes in the CLC membrane transporter (100 kDa) by using a monomeric ClC-ec1 variant (50 kDa) [125]. Solution NMR has also contributed to the characterization of many integral membrane proteins [126]. These include Human voltage-dependent anion channel (VDAC-1) [127], Bacterial outer membrane protein G [128] and mitochondrial uncoupling protein 2 [129]
- Solid state NMR on the other hand, uses quick sample spinning or alignment to produce excellent resolution in membrane proteins [130]. One of the main areas where solid state NMR exceeds solution NMR is that ssNMR have no limitation on the size of the protein [131]. For instance, ssNMR has allowed the study of the structure and dynamic of BAM complex (200 kDa) in lipid bilayer [132].

Solid state NMR is in particular significance in the study of membrane proteins. This is due to its ability to preserve the anisotropic nature of nuclear spin interactions, which is crucial for studying these proteins embedded within a lipid bilayer. The immobilization of polypeptides within the lipid bilayer timeframe aligns with the chemical shift and dipolar coupling spin interactions [113]. However, solely focusing on the studying of a protein’s anisotropic interactions is insufficient to gain a comprehensive understanding. To address the intrinsic anisotropic challenges of solid-state NMR, the application of magic angle spinning (MAS) has emerged. Through MAS, a combination of both isotropic and anisotropic interactions can be explored [133]. This approach facilitates the study of a protein’s structure within its native environment, capturing lipid-protein interactions, alongside the investigation of dynamic processes like conformational changes and ligand binding in membrane proteins. This results in enhanced resolution and sensitivity [134]. To illustrate this, similar to cryo-EM, MAS ssNMR has been used to study the structure and interactions of numerous GPCRs like rhodopsin, neuropeptide Y receptor and β2-adrenergic receptor [135].

For improved clarity and understanding, we’ve consolidated the information from this section into Table 2 presented below.

### 2.4. Biophysical Techniques

Many other biophysical techniques are widely used to study the structure of proteins. In this section we will discuss the use of nanodiscs, Atomic Force Microscopy, Neutron scattering, and patch clamp techniques in the characterization of membrane proteins. 

#### 2.4.1. Nanodiscs

Understanding the structure and function of membrane proteins is best achieved if studied within native-like environments [139] since the traditional use of detergents for the purification process has been shown to affect the normal folding, the stability, and the interaction of the proteins with their surroundings [140]. In an attempt to overcome the drawbacks of detergent use and to mimic the lipid bilayer’s natural environment, lipid nano discs are recently being employed in the study of membrane proteins [139]. These naondiscs take advantage of the hydrophobic properties of the human high density lipoprotein molecules HDL that are naturally found in the human lipid bilayer, and the amphipathic properties of the helical human apolipoprotein-A1 [141]. The engineered amphipathic protein creates a membrane scaffold protein MSP, looking like two belts wrapping around a disk shape lipid bilayer via its hydrophobic amino acids, and exposing its hydrophilic amino acids to the outside [139]. This allows the nanodisc to be suspended in a solution while holding the protein in a native-like environment, which preserves their natural folding, and therefore their active form [140]. In addition, this technique proves to tolerate a wide range of temperatures, to resist vigorous shaking, and to provide a transparent solution of thin consistency. It has shown resolution improvements in techniques such as NMR spectroscopy and cryo-EM. For example, Nanodics and cryo-EM have been used in the study of the ABC phospholipid transporter and in identification of the structure pf the 3a ion channel as part of the studies done for SARS-CoV-2. NMR also took advantage of the nanosdiscs in multiple studies such as the study of the interaction between cytochrome P450 with cytochrome b5 and NADPH in a lipid environment free of any detergents [141]. 

#### 2.4.2. Patch Clamp

As stated previously, channelopathies constitute the major cause of several diseases. Understanding the function of ion channels is crucial for understanding the physiological mechanism underlying many diseases and for developing appropriate drugs used in the treatment of channel-related diseases and other diseases as well [142]. Patch clamp has been considered the major technique for the study of the flow of ions through channels [142]. In this technique, a glass pipette with a narrow tip is attached and stabilized on a cell membrane containing ion channels while ensuring tight contact. Then a suction force is applied so that no ion can pass between the membrane and the pipette. When the channel opens, the ions pass into the pipette and the flow is measured using an electronic amplifier connected to the pipette [143]. This is the cell attached recording method. Other methods exists such as whole cell patch and perforated vesicle which disrupts the membrane studied, Outside-Out and Perforated Vesicle which uses solutions and investigate the effect of drugs, Inside-Out Patch which records the cytoplasmic side of the membrane, and the Loose Patch that is used in cases where the attachment between the pipette and the membrane cannot be done tightly [143]. However, the most advanced patch clamp method is the automated patch clamp which is considered a revolution in the field [144]. Instead of using one pipette and applying it on a small portion of a cell membrane, APC uses cell suspensions and planar multi-reading recording chip [144]. This allows multiple high-resolution recordings to be done simultaneously [144]. This technique offers several advantages for research in their studies of ion channels. It allows the researchers to change the intracellular solution while proceeding with recording simultaneously, to work at a wide range of temperatures, to record fast ion flows, to reduce the errors and noise induced using the traditional glass pipettes, and to average the recordings detected from multiple cells, which overall improves the accuracy of the measurements [144]. Automated patch clamp has been used in the study of multiple channels such as TRP, voltage gated sodium channels, glycine receptors among many others [144], as well as in the evaluation of new pharmaceutical products such as the safety of cardiac drugs on the heart [142].

#### 2.4.3. Atomic Force Microscopy

Atomic Force Microscopy (AFM) technique has several advantages such as, it can be applied to visualize large proteins complexes at various conformational states [145], as well as a simple sample preparation which can be used in air and liquid suspensions [146]. Regarding the structure of membrane proteins, AFM, which provides high-resolution imaging and probing capabilities, is becoming a useful technique [147]. The importance of AFM in clarifying the structural and functional characteristics of membrane proteins is currently being brought into focus in research. AFM has been used, for instance, to examine the conformational changes that membrane proteins undergo upon ligand binding and provide details about their allosteric processes [148]. Further, the investigation of protein-protein and protein-lipid interactions has been rendered available by AFM-based single-molecule force spectroscopy, offering knowledge about the dynamic behavior and stability of membrane protein complexes [149]. The nanomechanical properties of membrane proteins have also been studied using AFM, indicating their elasticity, flexibility, and response to mechanical stress [150]. These most recent improvements in AFM-based methods have assisted in a deeper comprehension of the structure-function relationship of membrane proteins and are extremely promising for further research in the area.

#### 2.4.4. Neutron Scattering

Neutron scattering can be enhanced to study different aspects of the proteins [151]. Techniques for neutron scattering have become effective methods for analyzing membrane proteins and provide novel insights into their dynamics. It permits to understand how membrane proteins behave in their natural lipid context, according to recent studies. In order to better understand the structure, interfacial characteristics, and interactions between proteins and lipids in lipid bilayers, neutron reflectometry has been used. The general shape, size, and oligomeric state of membrane proteins have been determined using small-angle neutron scattering (SANS) [152], providing an understanding of how they are assembled and organized structurally. In addition, the dynamics and conformational changes of membrane proteins have been studied using quasi-elastic neutron scattering (QENS) and neutron spin-echo spectroscopy (NSE) [153], as well as internal movements and domain dynamics. The structure-function connection of membrane proteins has recently been better understood because of recent developments in neutron scattering methods, which also show tremendous potential for further research in the area.

To enhance clarity and facilitate understanding, the information from this section has been consolidated into Table 3 below.

### 2.5. Computational Methods

Since each technique alone cannot give highly accurate data concerning the atomic positions, researchers have opted using computational methods in order to analyze different aspects of the protein. A combination of these methods needs to be used in order to clarify the structure and give a higher resolution 3D structure [159]. As a matter of fact, and in order to fully understand the structure, dynamics, and functional characteristics of membrane proteins, computational approaches have become necessary. Recent developments in computer approaches, such as molecular dynamics simulations, have made it possible to study the dynamics of membrane proteins at the atomic level, revealing information on their flexibility and conformational changes. When experimental approaches are challenging, the identification of membrane protein structures has been helped using homology modeling and de novo structure prediction algorithms. Furthermore, to examine protein-lipid interactions that plays crucial roles in the function of these proteins, GPCRs for example, and the organization of the membrane structure, molecular dynamics simulations proved insightful, revealing the precise binding sites and processes influencing membrane protein activity [160]. On the other hand, computational methods contribute to discovering protein ligands. First, structure modeling, along with ligand and structure-based methods, help in pinpointing potential ligands. Then, generating, and refining hit lists via database searches and ligand docking are integral steps in discovering ligands. For instance, since GPCR are extensively involved in cell signaling pathways, applying these computational methods in finding new GPCR ligands, opens the door for drug discovery [161]. Additionally, the prediction of membrane protein oligomerization has benefited from computational methods. Computational techniques that utilize known structural data on dimers, combined with others that employ quantum mechanical methods to study the chemical interactions between two GPCR monomers, yielded consistent and precise forecasts. These methods can help identify undiscovered GPCR dimers and enhance our comprehension and control of GPCR oligomers in biological contexts. Furthermore, these insights could assist experimental methods in determining GPCR oligomeric structures [162].

Softwares like phenix.refine (version 1.19.2-4158) [163], Qfit-3 (version 3.2.0) [164] and Refmac (version 5.7.0009) [165] were created so that researchers incorporate the huge amount of data collected from the mainly crystallographic methods such as X-ray crystallography and Cryo-EM and transform the data into virtual images [164,166]. In conclusion, our understanding of membrane protein structure and function has been greatly improved by the combination of experimental evidence and computer modeling.

## 3. Artificial Intelligence at the Service of Protein Structure

Artificial Intelligence (AI) is a field of computer science that aims to create intelligent machines that are capable of performing tasks normally requiring human intelligence, such as learning, problem-solving, and decision-making [167]. AI systems can be designed to carry out a wide range of tasks, including simple ones like recognizing patterns or sorting data, as well as more complex tasks like language translation [168]. Numerous industries, including healthcare, science, banking, transportation, and entertainment, have used AI in a variety of procedures. By automating processes, increasing productivity, and opening up new possibilities, AI has the ability to completely transform many facets of our life. The responsible and beneficial usage of AI technology, however, depends on a number of crucial elements, including ethical concerns, transparency, and responsible development.

### 3.1. Application of AI

There are various methods available for creating AI systems, and we will discuss a few of them. While decision tree systems offer a visual representation of decisions and their possible outcomes in the form of a tree structure, rule-based systems explicitly encode the rules that the system must obey [168]. Machine learning algorithms rely on statistical models and data, making them particularly well-suited for tasks that require adaptability and the ability to learn from the provided data [167]. Large datasets may be used to train these algorithms, which can subsequently be used to generate predictions or take action [168]. It proved unmatched superiority to human intelligence in many fields, including strategic games like Chess and Go, in addition to other decision making grounds [169]. Currently, it is evolving in a non precedented way, affecting our everyday lives in various aspects including labor and daily life activities [170]. The enormous jump in establishing self-driving vehicles exemplifies the potential of AI [171]. Among these fields, AI has particularly proven its value in biology and healthcare. Automated learning techniques are being applied in molecular biology to analyze tremendous amount of data and build databases [172]. Pharmaceutical industries have taken advantage of the analytic and predictive capabilities of machine learning to accelerate drug development by markedly increasing the efficiency of clinical trials, resulting from a better model, conduction, and analysis [173]. Genomics studies have also implemented deep learning algorithms to process and analyze huge amounts of intricate datasets [174].

### 3.2. AI Methods in Biology

One of the most promising implications of AI in Biology is the emergence and rapid development of AI systems, neural networks, accurately predicting protein structure from its corresponding amino acid sequence, such as AlphaFold, RoseTTAFold and ESMFold [175,176]. After its success at the Critical Assessment of Structure Prediction (CASP) CASP13 in 2018 [177] and further domination in CASP14 in 2020, DeepMind released AlphaFold2 source code to the public [178]. Numerous researchers have delved into the creation of neural networks as a result of the advancements in this field. As seen in CASP15, this has sped up the development of protein structure prediction tools. Additionally, the use of these AI systems in their study has helped hundreds of research publications [179].

#### 3.2.1. Alphafold2

Alphafold2 is trained on a large dataset of experimentally sequenced proteins, taking into consideration geometric, physical, and evolutionary constraints affecting protein folding. It runs on a complex system encompassing various steps in generating a prediction. One of these steps is the generation of multi-sequence alignments (MSA) between an unknown sequence and similar sequences from other organisms. In addition to that, it employs transformers, tools that recognize patterns, enabling the system to take into consideration interactions between distant amino acids. No key step was identified experimentally, but rather every step in the system contributes a little in producing an accurate prediction [178]. Alphafold2 predicted 98.5% of the human proteome with 58% of confident predictions and 36% of very high confidence, which is a remarkable step forward in the field, since experimentally determined structures consist of 17% of the whole human proteome [178].

#### 3.2.2. RoseTTAFold

RoseTTAFold modified Alphafold2 code, resulting in a neural network that takes into account three aspects simultaneously: the patterns present in protein sequences, the interactions between amino acids within a protein, and the potential three-dimensional structure of the protein. Alphafold2 made more accurate predictions than RoseTTAFold, despite RoseTTAFold accuracy. RoseTTAFold capacity to recognize and simulate multi-protein complexes was, however, one of its benefits [180]. This led DeepMind to release their own system precisely trained to predict multimeric protein structures, AlphaFold-Multimer, which successfully predicted 72% of homomeric interactions, of which, 36% are highly accurate, and 70% in heteromeric interactions, 26% predicted with high accuracy, with likelihood for improvements in the future [181].

#### 3.2.3. ESMFold

ESMFold model, which also took inspiration from Alphafold2, presents a system with a different approach, where for example a large language model and disregarded MSA generation are added. Hence, the required processing resources are drastically reduced, and the speed of short sequence prediction is boosted by almost 60 times. However, doing so meant compromising precision. The enhanced prediction speed was utilized to carry out comprehensive structural analysis of proteins in metagenomics on a large scale. 617 million structures predictions from countless microorganisms were made, of which 225 million structures were predicted with high confidence, including proteins distinct from any empirically determined structures, giving biologists insight into some of the most unknown proteins [175].

#### 3.2.4. Improvements

Although these AI systems have made great strides, they still need to be improved. One restriction imposed by GPU memory constraints on the size of protein complexes that may be predicted [182] may prevent broad use. Additionally, as the number of chains in the complex rises, accuracy tends to decline [182]. One massive disadvantage is its weakness in taking into consideration the effects exerted by the protein environment on its structure, especially the lipid bilayer. Although it excels in predicting isolated soluble proteins, it struggles in predicting membrane proteins [183]. Alphafold2 also struggles in performing some of its predictions, for example it cannot foresee uncommon conformations. Ligand interaction and the conformational change therefore induced, the effects of pre-trained model (PTM) on protein folding, in addition to intrinsically disordered proteins (IDPs) containing partly structured sequences, and effects of mutations are all limitations of alphafold2. In addition to that, it is unable to offer insight into protein dynamics and stability [184]. However, applying experimental techniques, such as NMR, along with alphafold2 would be especially valuable since they exhibit complementary characteristics that enhance each other’s strengths and compensate for each other’s weaknesses [184].

To enhance clarity and facilitate understanding, the information from this section has been consolidated into Table 4 below.

## 4. Conclusions

The study of membrane proteins, including their structure, function, and dynamics, is thoroughly summarized in the present work. In order to expand our understanding, this review examines several experimental and computational methods used to study membrane proteins and emphasizes the value of multidisciplinary approaches. This article aims to shed light on the complexities of membrane protein biology by reviewing a variety of experimental and computational methods. Overall, this insightful review highlights the need of researching membrane proteins in order to understand their functions in eukaryotic cells and is a useful resource for scientists trying to grasp the intricate workings of these vital biomolecules.

## Figures and Tables

**Table 1:** Table summarizing separation techniques.

Separation and Analysis				
Technique	Description	Advantages	Limitations	References
SDS-PAGE Sodium dodecyl-sulfate gel electrophoresis	Separation method allowing protein separation by mass	Straightforward and rapid Cost-efficient Visualized with staining Can be paired with immunoblotting for further analysis and identification	Does not preserve the protein in its native state Provides narrow data about structure and function Difficulties in studying hydrophobic proteins Unfit for the analysis of oligomerized protein states	[27,28,29,59,60,61]
2-Dimensional Electrophoresis (SDS-PAGE/IEF)	Technique combining SDS-PAGE and Isoelectric focusing for the separation based on pI and mass.	Used and tested vigorously Highly reproducible and precise High resolution separation Relies on two parameters Visualized with staining	Time consuming and demanding Proteins are in the denatured state Risk of hydrophobic protein aggregation at their pI Moderate yield with high relative loss during IEF & equilibration Less efficient for low abundance proteins	[34,35,62,63]
2-Dimensional Electrophoresis (16-BAC/SDS-PAGE)	This combines SDS-PAGE and the use of the 16-BAC cationic detergent with a separation based on charge and hydrophobicity	Conditions favor unstable proteins High solubility and recovery of hydrophobic proteins Compatible with low molecular mass proteins Minimal losses Highly reproducible High loading capacity	Longer duration of electrophoresis Decreased protein stacking Added steps and complexity The need for specialized equipment	[34,45,64,65]
Blue Native PAGE (BN-PAGE)	While preserving proteins’ native state, this protocol is used to study and isolate membrane proteins.	Simple with quick results Highly sensitive Requires little sample amounts Proteins are in their native state Permits study of dynamic protein states at high resolutions Can be used to test detergents for crystallization of membrane proteins Can be paired with other techniques such as in-gel, immune-detection, and mass spectrophotometry	Incompatible with fluorescence detection due to Coomassie blue dye Influenced by interactions between proteins, lipids, and detergent Limited resolution between protein complexes of similar molecular weight.	[47,48,66,67,68,69]
Capillary Electrophoresis	An analytical method separating charged proteins based on their electrical mobility.	Wide range analysis High resolution Short analysis time Limited consumption of samples Complex sample analysis May be coupled with miscellaneous detection methods	Low loading capacity Risk of: Adsorption to silica capillary inner wall which decreases efficiency Signal suppression or comigration in the case of complex systems Degradation of proteins due to high temperature	[70,71,72,73,74]
Free Flow Electrophoresis	This technique analyses a continuous stream of proteins on a channel with an electric field perpendicular to the flow.	Proteins are not denatured Fast continuous separation High separation efficiency Minimal sample loss Large sample volume processing	Complex setup strategies may impair shelf-life and efficiency Bubbles from electrolysis Limited by Joule heating	[55,75,76,77]

**Table 2:** Table summarizing characterization techniques.

Characterization and Conformation				
Technique	Description	Advantages	Limitations	References
Crystallography	Determines the structure of protein crystals using the diffraction patterns collected by X-rays, electrons, or neutrons.	Famous and well-established technique. Gives high-resolution structures Determines 3D structure at near atomic or atomic resolution. Neutron protein crystallography is used to identify the positions of hydrogen and deuterium.	Time-consuming and labor-intensive process. Difficulty in forming large, well-ordered crystals. Inability to visualize hydrogen atoms. Insufficient detection of chemical heterogeneity. Can’t visualize proteins in their native environment	[78,81,83,84,93,136]
Cryogenic electron microscopy (Cryo-EM)	Visualizes high-resolution protein structures by imaging frozen samples with an electron microscope.	Doesn’t require crystallization Visualize and determine the structure of a wide range of molecular masses. Preserves the native structure of the membrane protein. Distinguish heterogeneous complexes.	Limited resolution compared to X-ray crystallography. Requires purified protein samples and specific expertise in sample preparation. Computationally demanding to acquire a 3D structure	[96,101,103,104,137]
Nuclear Magnetic Resonance (NMR)	Studies the nuclei in the atoms of protein to determine molecular structure, dynamics, and interactions.	Acquires 3D structures of membrane proteins at atomic resolution in its native form. Provides information on conformational dynamics and flexibility of proteins. Can detect proteins in both solution and solid-state environments. Suitable for analyzing intrinsic disordered proteins.	Requires isotopically labeled protein samples. Limited to smaller protein sizes compared to cryo-EM and X-ray crystallography. Can study proteins in isotropic or an anisotropic environment depending on the type of NMR.	[113,115,121,122,123,138]

**Table 3:** Table summarizing biophysical techniques.

	Biophysical Innovation			
Nanodiscs	Solubilizes membrane proteins in aqueous media while keeping them in a native-like environment.	Suitable for the study of membrane proteins. Provides a lipid bilayer of known structure and composition. Solubilizes the proteins in a thin and transparent solution. Preserves the structure and function of the membrane proteins. Tolerates a wide range of temperatures. Tolerates vigorous shaking. Used in drug discoveries and in disease research and therapeutics.	Uses replicas of the membrane lipids and proteins Works In a nanoscale May not accommodate large proteins. Assembly and purification challenges Costly production and usage	[139,140,141]
Patch clamp	Studies ion channels by studying the flow of ions through it.	High resolution simultaneous recordings of ion flows. Works at a wide range of temperatures. Detects minute electrical currents. Reveals channel kinetics and properties. Records fast ion flows. Reduces the possibility of error. Used in drug development and screening.	Limited to the study of channels Equipment maintenance cost Requires special equipment. Invasive technique Risk of damaging the cell Prone to electrical noise	[142,143,144]
Atomic Force Microscopy (AFM)	Gives images and characterizes the surfaces of membrane proteins at the nanoscale by scanning a probe tip and measuring forces between the tip and sample.	Visualize large protein complexes in multiple conformational states in real time. Simple sample preparation and can be used in air and liquid suspensions. Provides high-resolution imaging. Enables investigation of protein-protein and protein-lipid interactions. Allows for studying the nanomechanical properties of membrane proteins.	Cannot provide detailed internal structural information. Sample preparation and imaging artifacts can affect accuracy. Requires specific expertise in operating the equipment. Limited availability of specialized AFM equipment in some research settings Limited ability to study dynamic processes in real-time.	[145,146,147,149,150,154,155]
Neutron Scattering	Uses a beam of neutrons to determine the atomic structure, composition, dynamics, and magnetic properties of membrane proteins.	Provide insights into the depth of different aspects of big proteins. Allows for studying membrane proteins in their natural lipid context. Able to study the dynamic and atomic position of hydrogen atoms Nondestructive technique	Requires specialized facilities and instrumentation, including a neutron source. Complex data analysis and interpretation. Relatively lower resolution compared to other techniques. Requires careful preparation of samples and contrast matching for optimal results.	[151,156,157,158]

**Table 4:** Table summarizing AI techniques.

Artificial Intelligence				
Technique	Description	Advantages	Limitations	References
RoseTTAFold	“three-track” neural network developed by Baker lab, to predict the 3D structure of proteins from their amino acid sequences	Accurate predictions Capacity to recognize and simulate multi-protein complexes	Limited ability to predict uncommon conformations Struggles with membrane proteins High computational power required	[180]
AlphaFold2	Deep learning-based AI system developed by DeepMind that accurately predicts the 3D structure of proteins from their amino acid sequences	Highly accurate predictions	Weak in considering protein’s environment Unable to predict uncommon conformations Limited insights into protein dynamics and stability High computational power required Poor ability to recognize and simulate multi-protein complexes	[178,184]
AlphaFold-Multimer	An Alphafold model trained to predict protein-protein complexes	Predicts multimeric protein structures accurately	Improvement potential Limited insights into protein dynamics and stability	[181]
ESMFold2	AI system developed by meta that predicts protein structures using a large language model trained on a massive dataset of protein sequences.	Faster prediction speed Enables large-scale analysis Lower computational power required	Lower precision Struggles with membrane proteins, limited insights into protein dynamics and stability	[175]

## Data Availability

Not applicable.

## References

[1] Pollock N.L., Lee S.C., Patel J.H., Gulamhussein A.A., Rothnie A.J. (2018). Structure and function of membrane proteins encapsulated in a polymer-bound lipid bilayer. Biochim. Biophys. Acta Biomembr..

[2] Harb F., Giudici-Orticoni M.T., Guiral M., Tinland B. (2016). Electrophoretic mobility of a monotopic membrane protein inserted into the top of supported lipid bilayers. Eur. Phys. J. E.

[3] Zhou C., Zheng Y., Zhou Y. (2004). Structure prediction of membrane proteins. Genom. Proteom. Bioinform..

[4] Shen H.H., Lithgow T., Martin L. (2013). Reconstitution of membrane proteins into model membranes: Seeking better ways to retain protein activities. Int. J. Mol. Sci..

[5] Vinothkumar K.R., Henderson R. (2010). Structures of membrane proteins. Q. Rev. Biophys..

[6] Leanza L., Managò A., Zoratti M., Gulbins E., Szabo I. (2016). Pharmacological targeting of ion channels for cancer therapy: In vivo evidences. Biochim. Biophys. Acta.

[7] Guan L. (2022). Structure and mechanism of membrane transporters. Sci. Rep..

[8] Askari A. (2019). The sodium pump and digitalis drugs: Dogmas and fallacies. Pharmacol. Res. Perspect..

[9] Zhang J., Li D., Yue X., Zhang M., Liu P., Li G. (2018). Colorimetric in situ assay of membrane-bound enzyme based on lipid bilayer inhibition of ion transport. Theranostics.

[10] Syrovatkina V., Alegre K.O., Dey R., Huang X.Y. (2016). Regulation, Signaling, and Physiological Functions of G-Proteins. J. Mol. Biol..

[11] Lolodi O., Wang Y.M., Wright W.C., Chen T. (2017). Differential Regulation of CYP3A4 and CYP3A5 and its Implication in Drug Discovery. Curr. Drug Metab..

[12] Harb F.F., Tinland B. (2020). Toward Electrophoretic Separation of Membrane Proteins in Supported n-Bilayers. ACS Omega.

[13] Yao Y., Ding Y., Tian Y., Opella S.J., Marassi F.M. (2013). Membrane protein structure determination: Back to the membrane. Methods Mol. Biol..

[14] Trivedi R., Nagarajaram H.A. (2022). Intrinsically Disordered Proteins: An Overview. Int. J. Mol. Sci..

[15] Jaakola V.P., Scalise M. (2019). Membrane Proteins: New Approaches to Probes, Technologies, and Drug Design, Part II. SLAS Discov..

[16] Das B.B., Park S.H., Opella S.J. (2015). Membrane protein structure from rotational diffusion. Biochim. Biophys. Acta.

[17] Yeagle P.L., Lee D.A. (2002). Membrane protein structure. Biochim. Biophys. Acta.

[18] Escribá P.V., González-Ros J.M., Goñi F.M., Kinnunen P.K., Vigh L., Sánchez-Magraner L., Fernández A.M., Busquets X., Horváth I., Barceló-Coblijn G. (2008). Membranes: A meeting point for lipids, proteins and therapies. J. Cell Mol. Med..

[19] Moraes I., Evans G., Sanchez-Weatherby J., Newstead S., Stewart P.D. (2014). Membrane protein structure determination—The next generation. Biochim. Biophys. Acta.

[20] Shinde Dipa V., Surati J. (2016). Review on: Electrophoresis: Method for Protein Separation. Pharma Sci. Monit..

[21] Ferrara S.D., Cecchetto G., Cecchi R., Favretto D., Grabherr S., Ishikawa T., Kondo T., Montisci M., Pfeiffer H., Bonati M.R. (2017). Back to the Future-Part 2. Post-mortem assessment and evolutionary role of the bio-medicolegal sciences. Int. J. Leg. Med..

[22] Boonham N., Glover R., Tomlinson J., Mumford R. (2008). Exploiting generic platform technologies for the detection and identification of plant pathogens. Sustainable Disease Management in a European Context.

[23] Burnham-Curtis M.K., Straughan D.J., Hamlin B.C., Draheim H.M., Gray Partin T.K., Wostenberg D.J. (2021). Wildlife Forensic Genetics and Biodiversity Conservation: The Intersection of Science, Species Management, and the Law. Wildlife Biodiversity Conservation: Multidisciplinary and Forensic Approaches.

[24] Basim E., BASIM H. (2001). Pulsed-field gel electrophoresis (PFGE) technique and its use in molecular biology. Turk. J. Biol..

[25] Melillo A. (2013). Applications of serum protein electrophoresis in exotic pet medicine. Vet. Clin. Exot. Anim. Pract..

[26] Zhang C., Woolfork A.G., Suh K., Ovbude S., Bi C., Elzoeiry M., Hage D.S. (2020). Clinical and pharmaceutical applications of affinity ligands in capillary electrophoresis: A review. J. Pharm. Biomed. Anal..

[27] Matsumoto H., Haniu H., Komori N. (2019). Determination of Protein Molecular Weights on SDS-PAGE. Methods Mol. Biol..

[28] Brunelle J.L., Green R. (2014). One-dimensional SDS-polyacrylamide gel electrophoresis (1D SDS-PAGE). Methods Enzym..

[29] Di Girolamo F., Ponzi M., Crescenzi M., Alessandroni J., Guadagni F. (2010). A simple and effective method to analyze membrane proteins by SDS-PAGE and MALDI mass spectrometry. Anticancer Res..

[30] Wang Z., Mim C. (2022). Optimizing purification of the peripheral membrane protein FAM92A1 fused to a modified spidroin tag. Protein Expr. Purif..

[31] Kotani N., Nakano T., Kuwahara R. (2022). Host cell membrane proteins located near SARS-CoV-2 spike protein attachment sites are identified using proximity labeling and proteomic analysis. J. Biol. Chem..

[32] Fan F., Wang J., Chen H., Wei L., Zhang Z. (2023). Isolation and protein MdtQ analysis of outer membrane vesicles released by carbapenem-resistant Klebsiella pneumoniae. Microb. Pathog..

[33] Jin Y., Zhang J., Manabe T., Tan W. (2019). Comparison of the performance of 1D SDS-PAGE with nondenaturing 2DE on the analysis of proteins from human bronchial smooth muscle cells using quantitative LC-MS/MS. J. Chromatogr. B Analyt. Technol. Biomed. Life Sci..

[34] Braun R.J., Kinkl N., Beer M., Ueffing M. (2007). Two-dimensional electrophoresis of membrane proteins. Anal. Bioanal. Chem..

[35] Meleady P. (2018). Two-Dimensional Gel Electrophoresis and 2D-DIGE. Methods Mol. Biol..

[36] Capdeville P., Martin L., Cholet S., Damont A., Audran M., Ericsson M., Fenaille F., Marchand A. (2021). Evaluation of erythropoietin biosimilars Epotin™, Hemax**^®^** and Jimaixin™ by electrophoretic methods used for doping control analysis and specific N-glycan analysis revealed structural differences from original epoetin alfa drug Eprex^®^. J. Pharm. Biomed. Anal..

[37] Molloy M.P., Herbert B.R., Slade M.B., Rabilloud T., Nouwens A.S., Williams K.L., Gooley A.A. (2000). Proteomic analysis of the Escherichia coli outer membrane. Eur. J. Biochem..

[38] Hamid N., Jain S. (2008). Characterization of an outer membrane protein of Salmonella enterica serovar Typhimurium that confers protection against typhoid. Clin. Vaccine Immunol..

[39] Kawai K., Liu Y., Ohnishi K., Oshima S.-i. (2004). A conserved 37 kDa outer membrane protein of Edwardsiella tarda is an effective vaccine candidate. Vaccine.

[40] Peng X., Ye X., Wang S. (2004). Identification of novel immunogenic proteins of Shigella flexneri 2a by proteomic methodologies. Vaccine.

[41] Cullen P.A., Cordwell S.J., Bulach D.M., Haake D.A., Adler B. (2002). Global analysis of outer membrane proteins from Leptospira interrogans serovar Lai. Infect. Immun..

[42] Hu Q., Ding C., Tu J., Wang X., Han X., Duan Y., Yu S. (2012). Immunoproteomics analysis of whole cell bacterial proteins of Riemerella anatipestifer. Vet. Microbiol..

[43] Zhang M.J., Gu Y.X., Di X., Zhao F., You Y.H., Meng F.L., Zhang J.Z. (2013). In Vitro Protein Expression Profile of Campylobacter jejuni Strain NCTC11168 by Two-dimensional Gel Electrophoresis and Mass Spectrometry. Biomed. Environ. Sci..

[44] Bednarz-Misa I., Serek P., Dudek B., Pawlak A., Bugla-Płoskońska G., Gamian A. (2014). Application of zwitterionic detergent to the solubilization of Klebsiella pneumoniae outer membrane proteins for two-dimensional gel electrophoresis. J. Microbiol. Methods.

[45] Smejkal G., Kakumanu S. (2019). Two-Dimensional 16-BAC/SDS Polyacrylamide Gel Electrophoresis of Mitochondrial Membrane Proteins. Methods Mol. Biol..

[46] Philipp S., Jakoby T., Tholey A., Janssen O., Leippe M., Gelhaus C. (2012). Cationic detergents enable the separation of membrane proteins of Plasmodium falciparum-infected erythrocytes by 2D gel electrophoresis. Electrophoresis.

[47] Wittig I., Braun H.P., Schägger H. (2006). Blue native PAGE. Nat. Protoc..

[48] Nickel C., Brylok T., Schwenkert S. (2016). In Vivo Radiolabeling of Arabidopsis Chloroplast Proteins and Separation of Thylakoid Membrane Complexes by Blue Native PAGE. Methods Mol. Biol..

[49] Moreno-Loshuertos R., Marco-Brualla J., Meade P., Soler-Agesta R., Enriquez J.A., Fernández-Silva P. (2023). How hot can mitochondria be? Incubation at temperatures above 43 °C induces the degradation of respiratory complexes and supercomplexes in intact cells and isolated mitochondria. Mitochondrion.

[50] Zheng Y., Gibb A.A., Xu H., Liu S., Hill B.G. (2023). The metabolic state of the heart regulates mitochondrial supercomplex abundance in mice. Redox Biol..

[51] Shallan A., Guijt R., Breadmore M. (2013). Capillary Electrophoresis: Basic Principles.

[52] Kustos T., Kustos I., Gonda E., Kocsis B., Szabó G., Kilár F. (2002). Capillary electrophoresis study of outer membrane proteins of Pseudomonas strains upon antibiotic treatment. J. Chromatogr. A.

[53] Danish A., Lee S.Y., Müller C.E. (2017). Quantification of green fluorescent protein-(GFP-) tagged membrane proteins by capillary gel electrophoresis. Analyst.

[54] Tani Y., Kaneta T. (2020). Indirect capillary electrophoresis immunoassay of membrane protein in extracellular vesicles. J. Chromatogr. A.

[55] Guo Q., Liu L., Yim W.C., Cushman J.C., Barkla B.J. (2021). Membrane Profiling by Free Flow Electrophoresis and SWATH-MS to Characterize Subcellular Compartment Proteomes in Mesembryanthemum crystallinum. Int. J. Mol. Sci..

[56] Eubel H., Lee C.P., Kuo J., Meyer E.H., Taylor N.L., Millar A.H. (2007). Free-flow electrophoresis for purification of plant mitochondria by surface charge. Plant J..

[57] De Michele R., McFarlane H.E., Parsons H.T., Meents M.J., Lao J., González Fernández-Niño S.M., Petzold C.J., Frommer W.B., Samuels A.L., Heazlewood J.L. (2016). Free-Flow Electrophoresis of Plasma Membrane Vesicles Enriched by Two-Phase Partitioning Enhances the Quality of the Proteome from Arabidopsis Seedlings. J. Proteome Res..

[58] Xie H., Yang Y., Xia C., Lee T.-C., Pu Q., Lan Y., Zhang Y. (2022). Diffusional microfluidics for protein analysis. TrAC Trends Anal. Chem..

[59] Fe C.d.l., Assunção P., Rosales R.S., Antunes T., Poveda J.B. (2006). Characterisation of protein and antigen variability among Mycoplasma mycoides subsp. mycoides (LC) and Mycoplasma agalactiae field strains by SDS-PAGE and immunoblotting. Vet. J..

[60] Pujol-Pina R., Vilaprinyó-Pascual S., Mazzucato R., Arcella A., Vilaseca M., Orozco M., Carulla N. (2015). SDS-PAGE analysis of Aβ oligomers is disserving research into Alzheimer’s disease: Appealing for ESI-IM-MS. Sci. Rep..

[61] Nowakowski A.B., Wobig W.J., Petering D.H. (2014). Native SDS-PAGE: High resolution electrophoretic separation of proteins with retention of native properties including bound metal ions. Metallomics.

[62] Rabilloud T., Chevallet M., Luche S., Lelong C. (2010). Two-dimensional gel electrophoresis in proteomics: Past, present and future. J. Proteom..

[63] Joshi K., Patil D., Patwardhan B., Chaguturu R. (2017). Chapter 9—Proteomics. Innovative Approaches in Drug Discovery.

[64] Macfarlane D.E. (1983). Use of benzyldimethyl-n-hexadecylammonium chloride (“16-BAC”), a cationic detergent, in an acidic polyacrylamide gel electrophoresis system to detect base labile protein methylation in intact cells. Anal. Biochem..

[65] Hartinger J., Stenius K., Högemann D., Jahn R. (1996). 16-BAC/SDS-PAGE: A two-dimensional gel electrophoresis system suitable for the separation of integral membrane proteins. Anal. Biochem..

[66] Reisinger V., Eichacker L.A. (2006). Analysis of membrane protein complexes by blue native PAGE. Proteomics.

[67] Ma J., Xia D. (2008). The use of blue native PAGE in the evaluation of membrane protein aggregation states for crystallization. J. Appl. Crystallogr..

[68] Reisinger V., Eichacker L.A. (2008). Solubilization of membrane protein complexes for blue native PAGE. J. Proteom..

[69] Weiland F., Zammit C.M., Reith F., Hoffmann P. (2014). High resolution two-dimensional electrophoresis of native proteins. Electrophoresis.

[70] Zhang Z., Zhang F., Liu Y. (2013). Recent Advances in Enhancing the Sensitivity and Resolution of Capillary Electrophoresis. J. Chromatogr. Sci..

[71] Chen D., McCool E.N., Yang Z., Shen X., Lubeckyj R.A., Xu T., Wang Q., Sun L. (2023). Recent advances (2019–2021) of capillary electrophoresis-mass spectrometry for multilevel proteomics. Mass Spectrom. Rev..

[72] Fonslow B.R., Yates J.R. (2009). Capillary electrophoresis applied to proteomic analysis. J. Sep. Sci..

[73] Masár M., Hradski J., Schmid M.G., Szucs R. (2020). Advantages and Pitfalls of Capillary Electrophoresis of Pharmaceutical Compounds and Their Enantiomers in Complex Samples: Comparison of Hydrodynamically Opened and Closed Systems. Int. J. Mol. Sci..

[74] Rabilloud T., Vaezzadeh A.R., Potier N., Lelong C., Leize-Wagner E., Chevallet M. (2009). Power and limitations of electrophoretic separations in proteomics strategies. Mass Spectrom. Rev..

[75] Eichacker L.A., Weber G., Sukop-Köppel U., Wildgruber R. (2015). Free flow electrophoresis for separation of native membrane protein complexes. Methods Mol. Biol..

[76] Novo P., Jender M., Dell’Aica M., Zahedi R.P., Janasek D. (2016). Free Flow Electrophoresis Separation of Proteins and DNA Using Microfluidics and Polycarbonate Membranes. Procedia Eng..

[77] Turgeon R.T., Bowser M.T. (2009). Micro free-flow electrophoresis: Theory and applications. Anal. Bioanal. Chem..

[78] Zheng H., Handing K.B., Zimmerman M.D., Shabalin I.G., Almo S.C., Minor W. (2015). X-ray crystallography over the past decade for novel drug discovery—Where are we heading next?. Expert Opin. Drug Discov..

[79] O’Dell W.B., Bodenheimer A.M., Meilleur F. (2016). Neutron protein crystallography: A complementary tool for locating hydrogens in proteins. Arch. Biochem. Biophys..

[80] Shi D., Nannenga B.L., Iadanza M.G., Gonen T. (2013). Three-dimensional electron crystallography of protein microcrystals. Elife.

[81] Srivastava A., Nagai T., Srivastava A., Miyashita O., Tama F. (2018). Role of Computational Methods in Going beyond X-ray Crystallography to Explore Protein Structure and Dynamics. Int. J. Mol. Sci..

[82] Smyth M.S., Martin J.H. (2000). X ray crystallography. Mol. Pathol..

[83] Kermani A.A. (2021). A guide to membrane protein X-ray crystallography. FEBS J..

[84] Kwan T.O.C., Axford D., Moraes I. (2020). Membrane protein crystallography in the era of modern structural biology. Biochem. Soc. Trans..

[85] Li D., Caffrey M. (2020). Structure and Functional Characterization of Membrane Integral Proteins in the Lipid Cubic Phase. J. Mol. Biol..

[86] Landau E.M., Rosenbusch J.P. (1996). Lipidic cubic phases: A novel concept for the crystallization of membrane proteins. Proc. Natl. Acad. Sci. USA.

[87] Kato H.E., Zhang F., Yizhar O., Ramakrishnan C., Nishizawa T., Hirata K., Ito J., Aita Y., Tsukazaki T., Hayashi S. (2012). Crystal structure of the channelrhodopsin light-gated cation channel. Nature.

[88] Garman E.F., Weik M. (2017). Radiation Damage in Macromolecular Crystallography. Methods Mol. Biol..

[89] Nass K. (2019). Radiation damage in protein crystallography at X-ray free-electron lasers. Acta Crystallogr. D Struct. Biol..

[90] Nass Kovacs G. (2021). Potential of X-ray free-electron lasers for challenging targets in structure-based drug discovery. Drug Discov. Today Technol..

[91] Nango E., Iwata S. (2023). Recent progress in membrane protein dynamics revealed by X-ray free electron lasers: Molecular movies of microbial rhodopsins. Curr. Opin. Struct. Biol..

[92] Maveyraud L., Mourey L. (2020). Protein X-ray Crystallography and Drug Discovery. Molecules.

[93] Schroder G.C., Meilleur F. (2021). Metalloprotein catalysis: Structural and mechanistic insights into oxidoreductases from neutron protein crystallography. Acta Crystallogr. D Struct. Biol..

[94] Gajdos L., Blakeley M.P., Haertlein M., Forsyth V.T., Devos J.M., Imberty A. (2022). Neutron crystallography reveals mechanisms used by Pseudomonas aeruginosa for host-cell binding. Nat. Commun..

[95] Lyumkis D. (2019). Challenges and opportunities in cryo-EM single-particle analysis. J. Biol. Chem..

[96] Murata K., Wolf M. (2018). Cryo-electron microscopy for structural analysis of dynamic biological macromolecules. Biochim. Biophys. Acta Gen. Subj..

[97] Yip K.M., Fischer N., Paknia E., Chari A., Stark H. (2020). Atomic-resolution protein structure determination by cryo-EM. Nature.

[98] Ho C.M., Li X., Lai M., Terwilliger T.C., Beck J.R., Wohlschlegel J., Goldberg D.E., Fitzpatrick A.W.P., Zhou Z.H. (2020). Bottom-up structural proteomics: CryoEM of protein complexes enriched from the cellular milieu. Nat. Methods.

[99] Chen D.D., Hao J., Shen C.H., Deng X.M., Yun C.H. (2022). Atomic resolution Cryo-EM structure of human proteasome activator PA28gamma. Int. J. Biol. Macromol..

[100] Nakane T., Kotecha A., Sente A., McMullan G., Masiulis S., Brown P., Grigoras I.T., Malinauskaite L., Malinauskas T., Miehling J. (2020). Single-particle cryo-EM at atomic resolution. Nature.

[101] Earl L.A., Falconieri V., Milne J.L., Subramaniam S. (2017). Cryo-EM: Beyond the microscope. Curr. Opin. Struct. Biol..

[102] Quade N., Boehringer D., Leibundgut M., van den Heuvel J., Ban N. (2015). Cryo-EM structure of Hepatitis C virus IRES bound to the human ribosome at 3.9-A resolution. Nat. Commun..

[103] Davis J.H., Tan Y.Z., Carragher B., Potter C.S., Lyumkis D., Williamson J.R. (2016). Modular Assembly of the Bacterial Large Ribosomal Subunit. Cell.

[104] Renaud J.P., Chari A., Ciferri C., Liu W.T., Remigy H.W., Stark H., Wiesmann C. (2018). Cryo-EM in drug discovery: Achievements, limitations and prospects. Nat. Rev. Drug Discov..

[105] Cai K., Zhang X., Bai X.C. (2022). Cryo-electron Microscopic Analysis of Single-Pass Transmembrane Receptors. Chem. Rev..

[106] Wentinck K., Gogou C., Meijer D.H. (2022). Putting on molecular weight: Enabling cryo-EM structure determination of sub-100-kDa proteins. Curr. Res. Struct. Biol..

[107] Safdari H.A., Pandey S., Shukla A.K., Dutta S. (2018). Illuminating GPCR Signaling by Cryo-EM. Trends Cell Biol..

[108] Gallo M., Defaus S., Andreu D. (2022). Disrupting GPCR Complexes with Smart Drug-like Peptides. Pharmaceutics.

[109] Klenotic P.A., Morgan C.E., Yu E.W. (2021). Cryo-EM as a tool to study bacterial efflux systems and the membrane proteome. Fac. Rev..

[110] Zhang Z., Lizer N., Wu Z., Morgan C.E., Yan Y., Zhang Q., Yu E.W. (2023). Cryo-Electron Microscopy Structures of a Campylobacter Multidrug Efflux Pump Reveal a Novel Mechanism of Drug Recognition and Resistance. Microbiol. Spectr..

[111] Huang C.Y., Draczkowski P., Wang Y.S., Chang C.Y., Chien Y.C., Cheng Y.H., Wu Y.M., Wang C.H., Chang Y.C., Chang Y.C. (2022). In situ structure and dynamics of an alphacoronavirus spike protein by cryo-ET and cryo-EM. Nat. Commun..

[112] Dunstone M.A., de Marco A. (2017). Cryo-electron tomography: An ideal method to study membrane-associated proteins. Philos. Trans. R Soc. Lond B Biol. Sci..

[113] Opella S.J. (2015). Solid-state NMR and membrane proteins. J. Magn. Reson..

[114] Speyer C.B., Baleja J.D. (2021). Use of nuclear magnetic resonance spectroscopy in diagnosis of inborn errors of metabolism. Emerg. Top. Life Sci..

[115] Patching S.G. (2015). Solid-state NMR structures of integral membrane proteins. Mol. Membr. Biol..

[116] Thoma J., Burmann B.M. (2020). High-Resolution In Situ NMR Spectroscopy of Bacterial Envelope Proteins in Outer Membrane Vesicles. Biochemistry.

[117] Puthenveetil R., Vinogradova O. (2019). Solution NMR: A powerful tool for structural and functional studies of membrane proteins in reconstituted environments. J. Biol. Chem..

[118] Weber F., Bohme J., Scheidt H.A., Grunder W., Rammelt S., Hacker M., Schulz-Siegmund M., Huster D. (2012). 31P and 13C solid-state NMR spectroscopy to study collagen synthesis and biomineralization in polymer-based bone implants. NMR Biomed..

[119] Henry G.D., Sykes B.D. (1990). Structure and dynamics of detergent-solubilized M13 coat protein (an integral membrane protein) determined by 13C and 15N nuclear magnetic resonance spectroscopy. Biochem. Cell Biol..

[120] Rose-Sperling D., Tran M.A., Lauth L.M., Goretzki B., Hellmich U.A. (2019). 19F NMR as a versatile tool to study membrane protein structure and dynamics. Biol. Chem..

[121] Cheng R.C., Maduke M. (2020). Expanding the membrane-protein NMR toolkit. Nat. Chem. Biol..

[122] Ahmed R., Forman-Kay J.D. (2022). NMR insights into dynamic, multivalent interactions of intrinsically disordered regions: From discrete complexes to condensates. Essays Biochem..

[123] Palmer A.G. (2004). NMR characterization of the dynamics of biomacromolecules. Chem. Rev..

[124] Chill J.H., Naider F. (2011). A solution NMR view of protein dynamics in the biological membrane. Curr. Opin. Struct. Biol..

[125] Abraham S.J., Cheng R.C., Chew T.A., Khantwal C.M., Liu C.W., Gong S., Nakamoto R.K., Maduke M. (2015). 13C NMR detects conformational change in the 100-kD membrane transporter ClC-ec1. J. Biomol. NMR.

[126] Danmaliki G.I., Hwang P.M. (2020). Solution NMR spectroscopy of membrane proteins. Biochim. Biophys. Acta Biomembr..

[127] Hiller S., Garces R.G., Malia T.J., Orekhov V.Y., Colombini M., Wagner G. (2008). Solution structure of the integral human membrane protein VDAC-1 in detergent micelles. Science.

[128] Liang B., Tamm L.K. (2007). Structure of outer membrane protein G by solution NMR spectroscopy. Proc. Natl. Acad. Sci. USA.

[129] Berardi M.J., Shih W.M., Harrison S.C., Chou J.J. (2011). Mitochondrial uncoupling protein 2 structure determined by NMR molecular fragment searching. Nature.

[130] Yeh V., Goode A., Bonev B.B. (2020). Membrane Protein Structure Determination and Characterisation by Solution and Solid-State NMR. Biology.

[131] Sun S., Han Y., Paramasivam S., Yan S., Siglin A.E., Williams J.C., Byeon I.J., Ahn J., Gronenborn A.M., Polenova T. (2012). Solid-state NMR spectroscopy of protein complexes. Methods Mol. Biol..

[132] Xiang S., Pinto C., Baldus M. (2022). Divide and Conquer: A Tailored Solid-state NMR Approach to Study Large Membrane Protein Complexes. Angew. Chem. Int. Ed. Engl..

[133] Gopinath T., Weber D., Wang S., Larsen E., Veglia G. (2021). Solid-State NMR of Membrane Proteins in Lipid Bilayers: To Spin or Not To Spin?. Acc. Chem. Res..

[134] Nishiyama Y., Hou G., Agarwal V., Su Y., Ramamoorthy A. (2023). Ultrafast Magic Angle Spinning Solid-State NMR Spectroscopy: Advances in Methodology and Applications. Chem. Rev..

[135] Chandler B., Todd L., Smith S.O. (2022). Magic angle spinning NMR of G protein-coupled receptors. Prog. Nucl. Magn. Reson. Spectrosc..

[136] Bucker R., Hogan-Lamarre P., Mehrabi P., Schulz E.C., Bultema L.A., Gevorkov Y., Brehm W., Yefanov O., Oberthur D., Kassier G.H. (2020). Serial protein crystallography in an electron microscope. Nat. Commun..

[137] Nogales E. (2016). The development of cryo-EM into a mainstream structural biology technique. Nat. Methods.

[138] Pham T.T.T., Rainey J.K. (2021). On-cell nuclear magnetic resonance spectroscopy to probe cell surface interactions. Biochem. Cell Biol..

[139] Chen A., Majdinasab E.J., Fiori M.C., Liang H., Altenberg G.A. (2020). Polymer-Encased Nanodiscs and Polymer Nanodiscs: New Platforms for Membrane Protein Research and Applications. Front. Bioeng. Biotechnol..

[140] Padmanabha Das K.M., Shih W.M., Wagner G., Nasr M.L. (2020). Large Nanodiscs: A Potential Game Changer in Structural Biology of Membrane Protein Complexes and Virus Entry. Front. Bioeng. Biotechnol..

[141] Sligar S.G., Denisov I.G. (2021). Nanodiscs: A toolkit for membrane protein science. Protein Sci..

[142] Bell D.C., Fermini B. (2021). Use of automated patch clamp in cardiac safety assessment: Past, present & future perspectives. J. Pharmacol. Toxicol. Methods.

[143] Leech C.A., Holz G.G.t. (1994). Application of patch clamp methods to the study of calcium currents and calcium channels. Methods Cell Biol..

[144] Bell D.C., Dallas M.L. (2018). Using automated patch clamp electrophysiology platforms in pain-related ion channel research: Insights from industry and academia. Br. J. Pharmacol..

[145] Ruggeri F.S., Sneideris T., Vendruscolo M., Knowles T.P.J. (2019). Atomic force microscopy for single molecule characterisation of protein aggregation. Arch. Biochem. Biophys..

[146] Pleshakova T.O., Bukharina N.S., Archakov A.I., Ivanov Y.D. (2018). Atomic Force Microscopy for Protein Detection and Their Physicosmall es, Cyrillichemical Characterization. Int. J. Mol. Sci..

[147] Fotiadis D. (2012). Atomic force microscopy for the study of membrane proteins. Curr. Opin. Biotechnol..

[148] Sanganna Gari R.R., Montalvo-Acosta J.J., Heath G.R., Jiang Y., Gao X., Nimigean C.M., Chipot C., Scheuring S. (2021). Correlation of membrane protein conformational and functional dynamics. Nat. Commun..

[149] Scholl Z.N., Marszalek P.E. (2018). AFM-Based Single-Molecule Force Spectroscopy of Proteins. Methods Mol. Biol..

[150] Medalsy I.D., Müller D.J. (2013). Nanomechanical Properties of Proteins and Membranes Depend on Loading Rate and Electrostatic Interactions. ACS Nano.

[151] Nakagawa H., Saio T., Nagao M., Inoue R., Sugiyama M., Ajito S., Tominaga T., Kawakita Y. (2021). Conformational dynamics of a multidomain protein by neutron scattering and computational analysis. Biophys. J..

[152] Wang T., Chen J., Du X., Feng G., Dai T., Li X., Liu D. (2022). How neutron scattering techniques benefit investigating structures and dynamics of monoclonal antibody. Biochim. Biophys. Acta Gen. Subj..

[153] Stingaciu L.R. (2022). Study of Protein Dynamics via Neutron Spin Echo Spectroscopy. J. Vis. Exp..

[154] Gołek F., Mazur P., Ryszka Z., Zuber S. (2014). AFM image artifacts. Appl. Surf. Sci..

[155] An Y., Manuguri S.S., Malmström J. (2020). Atomic Force Microscopy of Proteins. Methods Mol. Biol..

[156] Samarakoon A., Tennant D.A., Ye F., Zhang Q., Grigera S.A. (2022). Integration of machine learning with neutron scattering for the Hamiltonian tuning of spin ice under pressure. Commun. Mater..

[157] Hosseini M., Arif M., Keshavarz A., Iglauer S. (2021). Neutron scattering: A subsurface application review. Earth-Sci. Rev..

[158] Neumann D.A. (2006). Neutron scattering and hydrogenous materials. Mater. Today.

[159] Seffernick J.T., Lindert S. (2020). Hybrid methods for combined experimental and computational determination of protein structure. J. Chem. Phys..

[160] Tieleman D.P., Sejdiu B.I., Cino E.A., Smith P., Barreto-Ojeda E., Khan H.M., Corradi V. (2021). Insights into lipid-protein interactions from computer simulations. Biophys. Rev..

[161] Szwabowski G.L., Baker D.L., Parrill A.L. (2023). Application of computational methods for class A GPCR Ligand discovery. J. Mol. Graph. Model..

[162] Townsend-Nicholson A., Altwaijry N., Potterton A., Morao I., Heifetz A. (2019). Computational prediction of GPCR oligomerization. Curr. Opin. Struct. Biol..

[163] Logan D.T. (2020). Interactive model building in neutron macromolecular crystallography. Methods Enzym..

[164] Riley B.T., Wankowicz S.A., de Oliveira S.H.P., van Zundert G.C.P., Hogan D.W., Fraser J.S., Keedy D.A., van den Bedem H. (2021). qFit 3: Protein and ligand multiconformer modeling for X-ray crystallographic and single-particle cryo-EM density maps. Protein Sci..

[165] Yu J., Li S., Chen D., Liu D., Guo H., Yang C., Zhang W., Zhang L., Zhao G., Tu X. (2022). Crystal Structure of Human CD47 in Complex with Engineered SIRPα.D1(N80A). Molecules.

[166] Liebschner D., Afonine P.V., Baker M.L., Bunkóczi G., Chen V.B., Croll T.I., Hintze B., Hung L.W., Jain S., McCoy A.J. (2019). Macromolecular structure determination using X-rays, neutrons and electrons: Recent developments in Phenix. Acta Crystallogr. D Struct. Biol..

[167] Russell S.J., Norvig P. (2016). Artificial Intelligence: A Modern Approach.

[168] Goodfellow I., Bengio Y., Courville A. (2016). Deep Learning.

[169] Crandall J.W., Oudah M., Chenlinangjia T., Ishowo-Oloko F., Abdallah S., Bonnefon J.F., Cebrian M., Shariff A.F., Goodrich M.A., Rahwan I. (2017). Cooperating with machines. Nat. Commun..

[170] Feijóo C., Kwon Y., Bauer J.M., Bohlin E., Howell B., Jain R., Potgieter P., Vu K., Whalley J., Xia J. (2020). Harnessing artificial intelligence (AI) to increase wellbeing for all: The case for a new technology diplomacy. Telecomm. Policy.

[171] Biggi G., Stilgoe J. (2021). Artificial Intelligence in Self-Driving Cars Research and Innovation: A Scientometric and Bibliometric Analysis. Soc. Sci. Res. Netw..

[172] Rawlings C.J., Fox J.P. (1994). Artificial intelligence in molecular biology: A review and assessment. Philos. Trans. R Soc. Lond. B Biol. Sci..

[173] Kolluri S., Lin J., Liu R., Zhang Y., Zhang W. (2022). Machine Learning and Artificial Intelligence in Pharmaceutical Research and Development: A Review. AAPS J..

[174] Dias R., Torkamani A. (2019). Artificial intelligence in clinical and genomic diagnostics. Genome Med..

[175] Lin Z., Akin H., Rao R., Hie B., Zhu Z., Lu W., Smetanin N., Verkuil R., Kabeli O., Shmueli Y. (2023). Evolutionary-scale prediction of atomic-level protein structure with a language model. Science.

[176] Lee C., Su B.H., Tseng Y.J. (2022). Comparative studies of AlphaFold, RoseTTAFold and Modeller: A case study involving the use of G-protein-coupled receptors. Brief. Bioinform..

[177] AlQuraishi M. (2019). AlphaFold at CASP13. Bioinformatics.

[178] Jumper J., Evans R., Pritzel A., Green T., Figurnov M., Ronneberger O., Tunyasuvunakool K., Bates R., Zidek A., Potapenko A. (2021). Highly accurate protein structure prediction with AlphaFold. Nature.

[179] Elofsson A. (2023). Progress at protein structure prediction, as seen in CASP15. Curr. Opin. Struct. Biol..

[180] Baek M., DiMaio F., Anishchenko I., Dauparas J., Ovchinnikov S., Lee G.R., Wang J., Cong Q., Kinch L.N., Schaeffer R.D. (2021). Accurate prediction of protein structures and interactions using a three-track neural network. Science.

[181] Evans R., O’Neill M., Pritzel A., Antropova N., Senior A., Green T., Žídek A., Bates R., Blackwell S., Yim J. (2022). Protein complex prediction with AlphaFold-Multimer. bioRxiv.

[182] Bryant P., Pozzati G., Zhu W., Shenoy A., Kundrotas P., Elofsson A. (2022). Predicting the structure of large protein complexes using AlphaFold and Monte Carlo tree search. Nat. Commun..

[183] Azzaz F., Yahi N., Chahinian H., Fantini J. (2022). The Epigenetic Dimension of Protein Structure Is an Intrinsic Weakness of the AlphaFold Program. Biomolecules.

[184] Laurents D.V. (2022). AlphaFold 2 and NMR Spectroscopy: Partners to Understand Protein Structure, Dynamics and Function. Front. Mol. Biosci..

